# Adult meningitis in a setting of high HIV and TB prevalence: findings from 4961 suspected cases

**DOI:** 10.1186/1471-2334-10-67

**Published:** 2010-03-15

**Authors:** Joseph N Jarvis, Graeme Meintjes, Anthony Williams, Yolande Brown, Tom Crede, Thomas S Harrison

**Affiliations:** 1 Infectious Diseases Unit, GF Jooste Hospital, Cape Town, South Africa; 2 Desmond Tutu HIV Centre, Institute of Infectious Disease and Molecular Medicine, University of Cape Town, Cape Town, South Africa; 3 Centre for Infection, Department of Cellular and Molecular Medicine, St George's University of London, Cranmer Terrace, London SW17 0RE, UK; 4 Division of Infectious Diseases and HIV Medicine, Department of Medicine, University of Cape Town, Cape Town, South Africa; 5 Institute of Infectious Diseases and Molecular Medicine, University of Cape Town, Cape Town, South Africa

## Abstract

**Background:**

The presentation and causes of adult meningitis in South Africa have changed substantially as a result of HIV. Knowledge of aetiology and laboratory findings in patients presenting with meningitis are important in guiding management. We performed a retrospective study to determine these findings in a setting of high HIV and TB prevalence in Cape Town.

**Methods:**

Patients undergoing lumbar punctures between 1^st ^January 2006 and 31^st ^December 2008 at a public sector referral hospital were studied. Cases were classified by microbiological diagnosis, or in the absence of definitive microbiology as *1) *normal CSF (neutrophils ≤ 1 × 10^6^/L, lymphocytes ≤ 5 × 10^6^/L, protein ≤ 0.5 g/dL, glucose ≥1.5 mmol/L), *2) *minor abnormalities (neutrophils 2-5, lymphocytes 6-20, protein 0.51-1.0, glucose 1.0-1.49) or *3) *markedly abnormal (neutrophils>5, lymphocytes>20, protein>1.0, glucose<1.0).

**Results:**

5578 LPs were performed on 4549 patients, representing 4961 clinical episodes. Of these, 2293 had normal CSF and 931 had minor abnormalities and no aetiology identified. Of the remaining 1737, microbiological diagnoses were obtained in 820 (47%). Cryptococcus accounted for 63% (514) of microbiological diagnoses, TB for 28% (227), bacterial meningitis for 8% (68). Of the remaining 917 who had marked abnormalities, the majority (59%) had a sterile lymphocytic CSF. Of note 16% (81) patients with confirmed Cryptococcus, 5% (12) with TB and 4% (3) with bacterial meningitis had normal CSF cell-counts and biochemistry.

**Conclusions:**

Cryptococcal and tuberculous meningitis are now the commonest causes of adult meningitis in this setting. TB meningitis is probably underdiagnosed by laboratory investigation, as evidence by the large numbers presenting with sterile lymphocytic markedly abnormal CSFs.

## Background

South Africa has the highest burden of human immunodeficiency virus (HIV) disease in the world, with an estimated 5.5 million cases accounting for 17% of the global total [1]. This is associated with one of the world's highest tuberculosis (TB) incidence rates [1]. The presentation and causes of adult meningitis have changed substantially as a result of these ongoing epidemics [2,3]. Advanced HIV-infection can make interpretation of both clinical and laboratory findings difficult, hence knowledge of aetiology and laboratory findings in patients presenting with meningitis are important in guiding management. We performed a retrospective study to determine these findings in a setting of high HIV and tuberculosis prevalence in Cape Town.

## Methods

The study was approved by the Research Ethics Committee of the University of Cape Town, and performed at GF Jooste Hospital, a public sector adult referral hospital serving a population of 1.3 million. Forty-three percent of patients admitted to the medical wards have proven or clinically suspected HIV [4], and TB incidence in the referral area exceeds 1000/100,000 person-years [5].

### Participants and procedures

Patients undergoing lumbar-punctures (LPs) between 1^st ^January 2006 and 31^st ^December 2008 were studied. Results of all LPs performed for any indication were recorded, the majority being for suspected meningitis in acute admissions, with demographic information from laboratory request forms and clinical notes. Cerebrospinal-fluid (CSF) samples underwent macroscopic examination, protein and glucose quantification, cell counts using a Neubauer counting chamber following crystal violet staining, Gram-staining of centrifuged sediment, and bacterial culture on blood and chocolate agar for 72 hours. India-ink staining of centrifuged CSF, cryptococcal antigen (CrAg) testing (Meridian Cryptococcal Latex Agglutination System, Meridian Bioscience) if India-ink negative, and fungal culture of all samples on potato dextrose agar (PDA) slopes for 14 days were carried out. TB microscopy (Auramine-flourescent stain of the sediment when sufficient sample), liquid culture in mycobacterial growth indicator tubes (MGIT, Becton-Dickenson), Lowenstein-Jensen agar slopes if sufficient sample, and TB-PCR (Genotype MTBDRplus, Hain Lifesciences) on positive culture samples from MGIT were performed in cases of suspected TB meningitis (TBM) at the clinician's request, or when CSF findings were suggestive of TBM. In a limited number of cases TB-PCR was performed directly on CSF samples in an effort to rapidly obtain drug susceptibility data. VDRL and TPHA testing were carried out at the clinician's request.

### Evaluation and outcomes

When a patient had more than one LP, a separate clinical episode was defined when there was ≥1 month between LPs, except in cases of TBM, where any repeat LP within 6 months was considered part of the same episode. These definitions were based on the best available evidence, and aimed to reflect the total burden of meningitis due to differing aetiologies, including relapse episodes. Median duration of admission for cryptococcal meningitis at our hospital is 15 days, with an inter-quartile range (IQR) of 13 to 20 days (unpublished data). For TB meningitis a cut-of off 6 months was chosen, as patients are treated with 6-9 months of therapy, and the TB programme regards a re-presentation with TBM during treatment as a deterioration of the initial episode, rather than a "recurrence" or "relapse".

Cases were classified by microbiological diagnosis, or in the absence of definitive microbiology as:

*1) normal *CSF (neutrophils ≤ 1 × 10^6^/L, lymphocytes ≤ 5 × 10^6^/L, protein ≤ 0.5 g/dL, and glucose ≥1.5 mmol/L with no organism isolated),

*2) minor abnormalities *(neutrophils 2-5 × 10^6^/L, lymphocytes 6-20 × 10^6^/L, protein 0.51-1.0 g/dL, or glucose 1.0-1.49 mmol/L and no organism isolated) or

*3) markedly abnormal *(neutrophils>5 × 10^6^/L, lymphocytes>20 × 10^6^/L, protein>1.0 g/dL, or glucose<1.0 mmol/L and no organism isolated).

Markedly abnormal cases were further classified into *lymphocytic *(lymphocyte predominance, L_Ø_:N_Ø _ratio ≥3:1), *mixed *(L_Ø_:N_Ø _ratio between 3 and 0.33), and *pyogenic *(neutrophil predominance, N_Ø_:L_Ø _ratio ≥3:1).

HIV status was determined for all patients with microbiologically confirmed disease during the last 2 years of the study from laboratory records and clinical notes.

## Results

**Figure 1:**
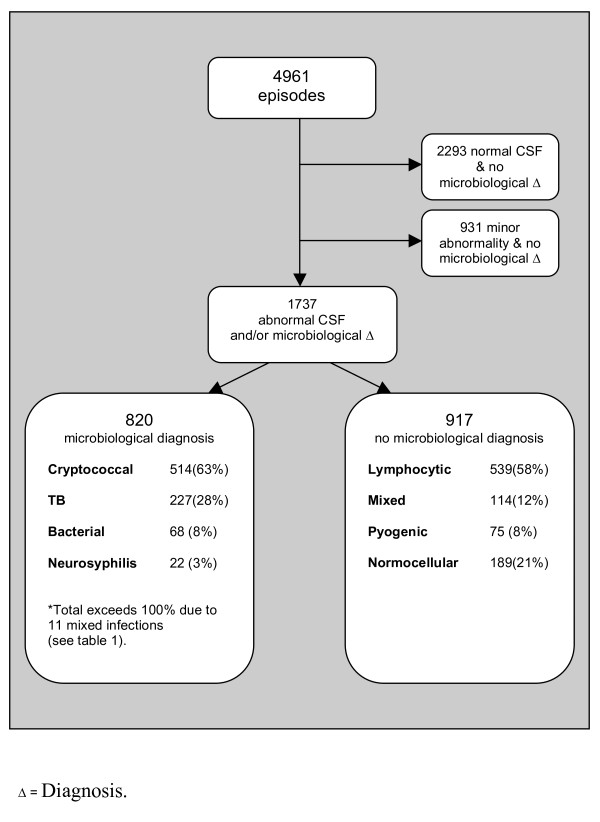
**CSF findings in the 4961 episodes, and aetiology of the 820 cases with a confirmed microbiological diagnosis**.

Over the 3-year study period 5578 LPs were performed on 4549 patients, representing 4961 clinical episodes. Forty-three percent of patients were male, and the median age was 34 years. Of these 4961 clinical episodes, 2293 had a normal CSF and no microbiological diagnosis. A further 931 episodes had minor abnormalities and no aetiology identified, compatible with the pleocytosis seen in HIV infection. Of the remaining 1737 episodes, microbiological diagnoses were obtained in 820 (47%). See figure 1.

### CSF findings

Cryptococcus (CM) accounted for 63% (514) of microbiological diagnoses, 93 (18%) of which were relapses of previously treated episodes. The CM relapse episodes occurred a median of 92 days after the initial episode (IQR 56-131 days). TB accounted for 28% (227), bacterial meningitis for 8% (68) and neurosyphillis for 3% (22)(figure 1). There were 2 patients who had 2 distinct episodes of TBM, one occurring 7 months and one occurring 10 months after the initial episode. CM was initially diagnosed on India-ink staining in 60% of cases, and CrAg testing in 40%. Cultures were positive in 68%. Laboratory confirmation of TBM was almost exclusively by culture, with smear positive microscopy in only 3 of the 227 cases. Of the culture confirmed bacterial meningitis, *Streptococcus pneumoniae *accounted for 90% (57 of 64), and *Neisseria meningitides *3% (2 of 64). Gram-staining was positive in 58 (85%) of the 68 patients.

Of the 917 cases with markedly abnormal CSF and no microbiological diagnosis, 539 (59%) had a lymphocytic predominance, 114 (12%) a mixed picture, 75 (8%) a polymorph predominance and 189 (21%) a normo-cellular picture with abnormal protein and/or glucose.

Of note 16% (81) patients with confirmed CM, 5% (12) with TBM and 4% (3) with bacterial meningitis had normal CSF cell counts, protein and glucose (table 1).

**Table 1:** Demographic information and laboratory findings in the 1737 cases with a microbiological diagnosis or markedly abnormal CSF.

	% * n = 1737	Age (years)	Sex (% Male)	Neutrophils (×10^6^/L)	Lymphocytes (×10^6^/L)	Protein (g/dL)	Glucose (mmol/L)	Normal CSF %(n)	HIV +ve %(n)	CD4 count (cells/μL)
*Cryptococcal*	30% (514)	34 (29-40)	50% (257)	0 (0-3)	18 (3-73)	0.97 (0.5-1.7)	2.1 (1.3-2.8)	16% (81)	99% (337/339)	39 (18-85)

*Tuberculous*	13% (227)	31 (27-40)	50% (116)	4 (0-25)	60 (16-157)	2.09 (1.2-3.8)	1.4 (0.8-2.3)	5% (12)	94% (126/134)	126 (61-201)

Bacterial**	4% (68)	33 (30-42)	34% (23)	29 (5-420)	46 (14-106)	5.0*** (2.7-5.0)	0.3*** (0.3-0.4)	4% (3)	97% (29/30)	287 (155-467).

*Neurosyphilis*	1% (22)	39 (26-44)	64% (14)	0 (0-1)	6 (1-64)	0.61 (0.4-1.0)	3.2 (2.5-3.6)	36% (8)	----------	----------

										

*Lymphocytic*	31% (539)	33 (27-41)	38% (206)	0 (0-4)	49 (27-123)	1.3 (0.7-2.3)	2.6 (2.1-3.4)	----------	----------	----------

*Mixed*	7% (114)	34 (28-44)	46% (53)	30 (10-124)	36 (17-114)	1.4 (0.7-2.9)	2.3 (0.9-2.9)	----------	----------	----------

*Pyogenic*	4% (75)	36 (27-42)	55% (41)	103 (14-824)	3 (1.6-3.7)	0.84 (0.5-2.6)	3 (1.6-3.7)	-----------	----------	----------

Normocellular****	11% (189)	35 (29-44)	49% (93)	0 (0-0)	1 (0-3)	1.5 (1.1-2.6)	3.0 (2.1-3.8)	----------	----------	----------

Definitions of lymphocytic, mixed, pyogenic and normo-cellular as defined in text.

All results are medians plus inter-quartile range unless otherwise stated.

*The denominator for the percentages expressed are the 1737 cases with markedly abnormal CSF cell counts, biochemistry and/or microbiological diagnoses. The cumulative percentage is over 100 as there were 11 mixed infections - 8 CM & TBM, 1 TBM & syphilis, 1 CM & syphilis, 1 CM and bacterial meningitis.

** 64 culture positive:*Streptococcus pneumoniae *90% (57 of 64), *Neisseria meningitides *3% (2 of 64), 1 × *β-haemolytic group A Streptococcus*, 1 × *Enterobacter cloacii*, 1 × *Streptococcus viridans*, 2 × *Staphlococcus aureus*. Four had gram stain only: 2 × gram negative diplococci, 2 × gram positive diplococci.

***Note the laboratory reported protein results up to 5.0 g/dL, and glucose results down to 0.3 mmol/L. Hence protein results of >5 g/dL and glucose results of <0.3 mmol/L were recorded as 5.0 and 0.3 respectively.

****Abnormal protein or glucose.

*Note CSF indices did not differ between initial and relapse episode*.

### HIV status

During the last two years of the study period HIV status was ascertained for all but 2 of the CM patients (n = 339), of whom 99% (337) were HIV positive, median CD4 cell count of 39 cells/μL; 134 of 158 TBM patients, 94% (126) of whom were HIV positive, median CD4 cell count 126 cells/μL; and 30 of the 46 bacterial meningitis cases, of whom 97% (29) were HIV positive, median CD4 cell count 287 cells/μL.

## Discussion

This is the largest reported series of adult meningitis in South Africa. CM and TBM are the commonest causes of adult meningitis in our setting (63% and 27% of microbiological diagnoses respectively). This is in marked contrast to the pre-HIV era [6,7], and in keeping with recent data from central and southern Africa showing that cryptococcosis is now the leading cause of adult meningitis, accounting for 27-45% of all cases [2,3,8-11]. As in these previously reported series, CM in our study was almost exclusively in the context of advanced HIV disease. Of note, during the study period approximately one third of initial episodes of CM occurred in patients already on ART [12], so that both initial and relapse episodes of CM were a mix of patients on and not on ART. This may explain why the proportion of cases diagnosed on India Ink and culture were lower, and those diagnosed by CrAg higher, than prior series, and also why CSF indices did not differ between initial and relapse episodes.

The proportion of TBM in our study was higher than in most reported cohorts from central and southern Africa, which range from 1-17% [2,3,8,10], however similar to figures from two small South African studies. TBM accounted for 25.4% of all adult meningitis in Soweto in 1996 [13], and increased from 16% in 1994 to 31% in 1998 in Pretoria [14]. The proportion of TBM in our study is almost certainly an under-estimate. TBM is under-diagnosed by routine laboratory investigation, with reported rates of microbiological confirmation ranging from 25 to 70% [15]. In a recent Vietnamese study, microbiological confirmation of TBM was obtained in 45% of HIV-positive patients, versus 33% of HIV-negative patients [16]. Assuming similar confirmation rates in our setting, it is likely that a substantial number of the 539 cases with no microbiological diagnosis, and lymphocytic CSF, represented TBM; an assumption supported by the very high community TB rates in our setting [5].

Further reasons for possible under-diagnosis of TBM were that not all CSF samples were sent for TB studies, volumes of CSF examined were low (typically 1-2 mls), and time for smear microscopy was limited. There is good evidence that diagnostic yields can be improved by collection of large CSF volumes for culture, and careful microscopic examination of centrifuged samples [17,18]. Smear positivity rates of 58-69% have been reported from Vietnam, where up to 10 mls of CSF was routinely obtained [17,18], and efforts are needed to improve the diagnosis of TBM in our setting.

Numbers of bacterial meningitis were relatively low. It is possible that prior antibiotic exposure hindered diagnosis in some cases, however numbers of sterile pyogenic CSFs were low. Numbers of bacterial meningitis appear to have stayed more or less constant despite the evolving HIV epidemic [14], but with a marked switch in aetiology. Meningococcus, which previously predominated, causing ~30% of adult bacterial meningitis [6,7], accounted for only 3% in our series, with Pneumococcus accounting for over 90% of bacterial meningitis.

The study highlights the fact that HIV co-infection, as well as changing the causative organisms, can lead to atypical laboratory presentations of meningitis. Substantial numbers of patients with CM, TB and bacterial meningitis had normal or nearly normal CSF findings. Of particular note were the findings in bacterial meningitis, almost all of which was HIV-associated. Four (6%) of 68 patients had acellular CSF, 30 (44%) of 68 had a lymphocyte predominance, and the majority had very elevated CSF protein. This adds to the diagnostic difficulties posed by the often overlapping clinical features of meningitis a population with a high HIV prevalence, further compounded by the presence of dual pathology in a small proportion of cases (table).

A limitation is that, as a laboratory based study, detailed clinical information was lacking. CM is unlikely to be missed, however, as discussed, TBM is likely to under-diagnosed by laboratory investigation, and this caveat must be taken into account when interpreting these data.

## Conclusions

The HIV and TB epidemics in South Africa have led to a marked change in the aetiology of adult meningitis. Cryptococcus has emerged as the dominant causative agent, the proportion of meningitis due to TB is increasing, and the spectrum of bacterial meningitis has been markedly altered. High rates of HIV in a population may make interpretation of CSF findings difficult, and it is important to take these data into account when investigating and managing patients with suspected meningitis. Mortality of both cryptococcal and TB meningitis remains high in this setting [11,18], and these results underscore the importance of continuing efforts to improve the early diagnosis and treatment of cryptococcal and TB meningitis.

## Competing interests

The authors declare that they have no competing interests.

## Authors' contributions

JNJ conceived and designed the study, collected and analysed the data and wrote the manuscript. GM and TSH assisted with study design, data analysis and manuscript preparation. AW and YB assisted with data collection and writing of the methods section. TC contributed to study conceptualization and critically reviewed the manuscript. All authors read and approved the final manuscript.

## Pre-publication history

The pre-publication history for this paper can be accessed here:

http://www.biomedcentral.com/1471-2334/10/67/prepub
